# Editorial: Knowledge and behavioral beliefs related to vaccination hesitancy among healthcare workers

**DOI:** 10.3389/fpubh.2024.1518112

**Published:** 2024-12-23

**Authors:** Divya S. Subramaniam, Ruaa Al Juboori, Aida Bianco, Dipti P. Subramaniam

**Affiliations:** ^1^Department of Health and Clinical Outcomes Research, Saint Louis University School of Medicine, St. Louis, MO, United States; ^2^Advanced HEAlth Data Institute, Saint Louis University, St. Louis, MO, United States; ^3^Department of Public Health, University of Mississippi School of Applied Sciences, Oxford, MS, United States; ^4^Department of Medical and Surgical Science, University of Magna Graecia, Catanzaro, Italy

**Keywords:** vaccination uptake, behavioral beliefs, health promotion, healthcare workers (HCWs), vaccination

## Healthcare workers' vaccine attitudes

Healthcare workers (HCW) are at an elevated risk of occupational exposure to various infectious diseases, thus making vaccination a key driver in reducing spread and transmission amongst their patients and within their healthcare settings (1). Research indicates that higher vaccination rates among HCWs can lead to reduced morbidity and mortality, therefore benefiting both patients and healthcare systems (2). On the contrary, another study found that skepticism about vaccine safety, fear of side effects, and distrust of pharmaceutical companies or public health initiatives can lead to vaccine refusal (3).

Given the significant role of the HCWs in promoting public trust in vaccine uptake, understanding their personal beliefs about vaccines is crucial. A study by Schmid et al. (4) found that that personal attitudes toward vaccination, perceived social norms, and trust in health authorities influence HCWs' willingness to be vaccinated. Research has also indicated that HCWs who are well-informed about vaccine safety and efficacy are more likely to get vaccinated and recommend vaccines to patients (5).

## Highlights from the Research Topic

Building on the importance of behavioral beliefs and their role in vaccination uptake, several studies provided insights into the challenges faced by HCWs in different contexts. Getachew et al. investigated COVID-19 vaccine acceptance among healthcare workers in Eastern Ethiopia, finding a low acceptance rate of 35.6%. Similarly, Asefa et al. study in the West Guji zone of Southern Ethiopia reported a slightly higher, but relatively low acceptance rate of 38.1% as well. These studies identified key factors influencing willingness to vaccine acceptance such as age, professional role, prior vaccine side effects, positive attitudes toward vaccination, perceptions of susceptibility and severity of the disease, and knowledge about the vaccines.

Furthermore, study findings underscore the grave need for government and stakeholder collaboration to increase vaccine awareness, address safety concerns, and dispel any misconceptions through targeted campaigns. Enhancing vaccine education and promoting preventive practices among HCWs will be essential to improve acceptance rates in these geographic regions. Another study by Polla et al. investigated HCWs willingness in Italy to receive a second COVID- 19 vaccination booster dose. It found that only 52.6% of HCWs were willing to receive the COVID-19 booster and was driven primarily by a desire to protect their family members and patients. Key factors influencing their willingness include beliefs about COVID-19's severity and the vaccine's overall effectiveness. This study emphasizes the need for targeted educational interventions to enhance vaccine uptake and encourage HCWs to recommend it to their patients.

Beyond vaccine hesitancy, challenges faced by HCWs during COVID-19 pandemic extend to issues of burnout. The article by Gu et al. examines factors contributing to burnout among Chinese vaccination staff during the COVID-19 pandemic. This study identified key elements such as workload, emotional exhaustion, and support from colleagues as significant contributors to burnout levels. These findings suggest that addressing these factors through improved organizational support and mental health resources could mitigate burnout among vaccination staff, ultimately enhancing their wellbeing and effectiveness.

Expanding on the role of HCWs beliefs and behaviors in vaccination uptake, several studies examined vaccination patterns beyond COVID-19. The cross-sectional study by Mercogliana et al. explores tetanus, diphtheria, and pertussis (Tdap) booster vaccination among healthcare workers (HCWs) in a large academic hospital in Southern Italy. This study found that only 34.5% of HCWs had received the booster in the past 10 years. Factors such as job seniority influenced vaccination rates, with those employed for 5–9 years being less likely to receive it. Study findings highlight the need for targeted public health strategies to increase vaccine awareness and uptake, especially in high-risk healthcare settings.

Similarly, a study by Licata et al. analyzes pertussis vaccination among pregnant women in Italy by surveying HCWs like OB-GYNs, midwives, and primary care physicians. Although, most HCWs had good knowledge of the vaccine, their recommendation practices varied. Those with higher awareness of the vaccine's effectiveness were more likely to promote it. Midwives and primary care physicians were less likely to recommend vaccination, citing reasons like vaccine hesitancy and lack of knowledge. These findings highlight the importance of improved education and strategies to boost vaccine uptake among HCWs and their patients.

Fan et al. systematic review shift focuses to influenza vaccination revealing a global HCW vaccination rate of 41.7%. Furthermore, vaccination rates varied by region, with the highest in the Americas (67.1%) and the lowest in Africa (6.5%). Factors influencing vaccination uptake include age, education, length of service, awareness of risks, and belief in vaccine efficacy. This review calls for comprehensive strategies to promote flu vaccination, especially in regions with lower rates, and highlights the need for targeted interventions to improve uptake among HCWs.

Pouvrasseau and Jeannot's study offers insights into vaccine hesitancy among nursing and midwifery students in Switzerland, particularly focusing on the HPV vaccine. Using an online questionnaire, the study assesses students' general vaccine confidence, HPV vaccination rates, and willingness to recommend the HPV vaccine. It also explores factors such as socio- demographic characteristics and interest in complementary medicine. These findings highlight the need for targeted educational strategies to improve vaccine confidence among future healthcare professionals, ensuring better public health outcomes.

In the multicenter study In Istanbul, Turkey by Parlak et al., explores the perspectives of pediatricians, gynecologists, nurses, and mothers regarding the human papillomavirus (HPV) vaccine. It highlights the importance of healthcare professionals' recommendations in influencing mothers' attitudes toward HPV vaccination for their daughters. This study identifies barriers to vaccination, including lack of awareness and misconceptions about the vaccine's safety and efficacy. The authors emphasize the need for improved communication strategies among healthcare providers to enhance vaccination rates and protect against HPV-related diseases.

In the commentary by Finsterer, the author critiques a study that assessed the quality of life (QoL) of post-hospitalization COVID-19 patients 1 year following infection. Moreover, the author highlights methodological limitations, such as the use of telephone interviews and the generality of the SF-36 QoL questionnaire, which may not fully capture the specific long-term effects of COVID-19. The author advocates for more comprehensive assessments, including in- person evaluations and targeted questions about COVID-19 symptoms and vaccination impacts, to improve understanding of patient health outcomes.

## Conclusion

Research on vaccine acceptance among HCWs reveals a complex mix of factors influencing vaccination uptake and decisions. Low uptake of vaccines such as COVID-19, Tdap, and HPV highlights existing barriers that require urgent attention. Future research to improve vaccine acceptance among HCWs must focus on developing targeted educational and behavioral interventions that address specific misconceptions and knowledge gaps. Lastly, longitudinal research studies are needed to assess the effectiveness and long-term impact of interventions on vaccination uptake and to further identify evolving factors influencing vaccination acceptance.
